# Habit Reversal Training and Variants of Decoupling for Use in Body-Focused Repetitive Behaviors. A Randomized Controlled Trial

**DOI:** 10.1007/s10608-022-10334-9

**Published:** 2022-11-17

**Authors:** Steffen Moritz, Danielle Penney, Alina Bruhns, Sarah Weidinger, Stella Schmotz

**Affiliations:** 1 Department of Psychiatry and Psychotherapy, University Medical Center Hamburg-Eppendorf, Hamburg, Germany; 2 Centre Intégré Universitaire de Santé et de Services Sociaux de l’Ouest-de-l’Île-de-Montréal Douglas Mental Health University Institute, Verdun, Canada

**Keywords:** Habit reversal training, Decoupling, Body-focused repetitive behaviors, Trichotillomania, Self-help, Skin picking

## Abstract

**Background:**

Behavioral interventions hold promise in improving body-focused repetitive behaviors (BFRBs), such as hair pulling and skin picking. The effect of combining different treatment techniques is currently unknown.

**Methods:**

In the framework of a randomized controlled crossover trial, 334 individuals with at least one BFRB were allocated either to a waitlist control or to three experimental conditions (1:1:1:1). Participants in the experimental condition received self-help manuals teaching habit reversal training (HRT), decoupling (DC) and decoupling in sensu (DC-is) during a six-week period. Treatment conditions differed only in the order of manual presentation. We examined whether applying more than one technique would lead either to add-on or interference effects.

**Results:**

The three treatment conditions were significantly superior to the waitlist control group in the improvement of BFRBs according to intention-to-treat analyses at a medium effect size (all *p* ≤ 0.002, *d* = 0.52 – 0.54). The condition displaying DC first significantly reduced depressive symptoms (*p* = 0.003, *d* = 0.47) and improved quality of life (*p* = 0.011, *d* = 0.39) compared to the waitlist control. Those using more techniques concurrently showed the strongest decline in BFRB symptoms, even after controlling for days practiced. Participants rated all manuals favorably, with standard DC and HRT yielding greatest acceptability.

**Discussion:**

Results tentatively suggest the concurrent application of different behavioral treatments for BFRBs leads to add-on effects. Results were superior when DC was practiced first, with positive effects extending to depressive symptoms and quality of life. Integrating the three techniques into one self-help manual or video along with other treatment procedures (e.g., stimulus control techniques) is recommended.

## Introduction

### Subtypes of Body-Focused Repetitive Behaviors (BFRBs)

Body-focused repetitive behaviors (BFRBs) represent a complex syndrome that can be described as an excessive/dysfunctional variant of primitive grooming behavior, also seen in animals (D’Angelo et al., 2014; Maraz et al., 2017). While only trichotillomania (i.e., urge resulting in pulling one’s hair) and skin picking (i.e., urge resulting in picking one’s skin) are listed as disorders in the DSM-5, nail biting (i.e., urge resulting in biting or gnawing one’s fingernails), dermatophagia (i.e., urge resulting in biting/eating one’s skin) and lip-cheek biting (LCB; urge resulting in biting the inside of one’s cheek, lip or tongue) are also counted as BFRBs. BFRBs remain under-diagnosed, under-treated and under-researched despite their high prevalence, which is as high as 90% for occasional BFRBs and up to 10% for excessive BFRBs (Houghton et al., 2018; Sampaio & Grant, 2018). This study considered BFRBs more generally, and included participants with subclinical (non-pathological) forms of the disorder. In turn, the term body-focused repetitive behavior disorder (BFRBD) should be reserved for those meeting full diagnostic criteria confirmed by a clinician. Of note, BFRBs do not only involve severe psychological consequences, such as low self-esteem, feelings of shame and guilt, comorbid depression or reduced quality of life, but result in social and occupational impairments across its different forms and also in those who do not meet full diagnostic criteria (Flessner & Woods, 2006; Keuthen et al., 2000; Solley & Turner, 2018; Wetterneck et al., 2006). Some conditions, such as skin picking, have also been associated with suicidal behavior (Machado et al., 2018). Furthermore, the physical consequences of BFRBs can be very serious: Affected individuals can suffer from bald patches and visible scars (Flessner & Woods, 2006; Grant et al., 2012). BFRBs may even lead to life-threatening sequelae such as large wounds (Bain & Vincent, 2016) that may lead to sepsis in skin picking or bezoars in trichotillomania/-phagia (Chin & Ng, 2021).

### Problems Relating to Nomenclature, Boundaries/Differential Diagnosis and Definition

The reasons for under-diagnosing BFRBs are multifaceted; different BFRBs are scattered across psychiatric and somatic classification systems, burdening scientific visibility and research designs. Thus far, only trichotillomania (i.e., 312.39) and skin picking (i.e., 698.4) have DSM codes (American Psychiatric Association, 2013).

The boundaries regarding which behaviors to include as BFRBs are also unclear. While trichotillomania, skin picking, LCB and nail biting are mentioned in the DSM-5, dermatophagia, awake bruxism (i.e., teeth grinding while awake) and joint cracking are not, despite being regarded as BFRBs by most researchers (e.g., Houghton et al., 2018; Woods & Houghton, 2015). These conditions will also be considered BFRBs in the present study.

The term BFRB is itself prone to misunderstanding; strictly speaking, eating, drinking, walking and breathing are also body-focused repetitive behaviors and the repetitive character (i.e., being performed in ritualized fashion) does not apply to all of its manifestations (Moritz et al., 2012). While some BFRBs relate to a circumscribed body region that is targeted in a stereotypal fashion (e.g., pulling out one’s eyelashes), other BFRBs, especially skin-picking, may have a more (sensory-driven) bottom-up nature with different locations and involving different muscle innervations and modes of action (e.g., fingers, instruments), depending on the core condition (Arnold et al., 1998; Moritz et al., 2012; Tucker et al., 2011). Finally, it remains controversial whether BFRBs are best subsumed under obsessive-compulsive and related disorders (see DSM-5), as unlike in obsessive-compulsive disorder (OCD), no obsessive thought precedes BFRBs. More importantly, and unlike compulsions, BFRBs are the execution of an urge and not its prevention or “displacement”, as in OCD.

### Pharmacological, Psychological and Self-Help Treatment for BFRBs

No psychopharmacological agent is currently approved for the treatment of BFRB. Yet, off-label use of antidepressants, naltrexone, or N-acetylcysteine is common, especially in trichotillomania, for which the most trials have been conducted. A meta-analysis cautions that small sample sizes, low statistical power, and single-center study trials may in part account for potentially inflated response rates (Baczynski & Sharma, 2020, p. 7). Some individuals seem to benefit from antipsychotic medication, although these agents should be contemplated as a last resort due to a lack of controlled studies and frequent adverse events (Jones et al., 2018) that may in fact worsen some BFRBs (e.g., LCB due to tardive dyskinesia). Moreover, the occasional benefits of pharmacological agents seem to be eclipsed by the efficacy of HRT (Farhat et al., 2020).

Several behavioral techniques exist for the treatment of BFRBs (please see "Methods" section). Habit reversal training (HRT; Azrin & Nunn, 1973) has the largest evidence base with multiple studies showing its benefit (e.g., Bate et al., 2011; Lee et al., 2019; Lochner et al., 2017). In HRT, a competing response is executed via an antagonist tensing of the muscles (i.e., a freezing behavior) – usually lasting up to 3 min. HRT consists of multiple components; competing response training and awareness training represent its most effective techniques (Miltenberger et al., 1985). Promising results have also been reported for dialectical behavior therapy (DBT)-enhanced HRT (Keuthen et al., 2011), comprehensive behavioral treatment (COMB; Carlson et al., 2021) and acceptance and commitment therapy (ACT; Lee et al., 2020). HRT-based self-help techniques have been developed more recently as a response to the treatment gap, due to factors such as shame among affected individuals or lack of specialized therapists. Decoupling (DC; Moritz & Rufer, 2011) is another technique showing efficacy in controlled trials (for a systematic review see Lee et al., 2019). In DC, a behavior that mimics the old (dysfunctional) behavioral path must be performed first. In close proximity to the conventional target (e.g., next to the mouth in the case of nail biting) the behavior is then diverted (using an accelerated movement) in another direction in order to deviate from and replace the dysfunctional behavior. The latest technique, decoupling in sensu (DC-is), was first examined in an individual with dermatophagia (Moritz & Rufer et al., 2020). During DC-is, the first part of the DC technique is performed exclusively in the imagination, which is hoped to facilitate generalization in the event the dysfunctional behavior relates to multiple locations (Moritz et al., 2012). When directly comparing the three techniques (Moritz et al., 2021), 34.8% of completers in the DC group showed an improvement of at least 35% on the generic body-focused repetitive behavior scale (GBS) compared to 10.0% in the HRT, and 23.3% in the DC-is groups. A dose-effect relationship emerged (particularly for HRT) which suggests that applying the techniques more frequently may increase effects. Subjective appraisal ratings were more favorable for DC-is and HRT than for DC.

### The Present Study

The present study was planned as a crossover trial wherein participants with at least one BFRB were allocated to four groups: a waitlist control and three treatment groups. The treatment groups received three manuals conveying the behavioral interventions: HRT, DC and DC-is. The experimental groups only differed with respect to the order of manual delivery, in an attempt to balance primacy versus recency effects, as the first and last manual may have a processing advantage (e.g., no interference for the first manual; better recollection of the last manual). Psychological treatment programs, such as behavior therapy, usually encompass multiple elements/techniques and it remains unknown in most cases whether order effects play a role, that is, the sequence by which different techniques are either conveyed by the therapist or exercises performed by the patient. By balancing for order, we could examine whether the sequence would influence results.

The study assessed, with respect to BFRB and depressive symptoms, (1) whether the parallel or subsequent application of the manuals would yield surplus effects or (2) whether the application of different manuals is redundant (e.g., due to potentially similar modes of action), or if the combined application leads to interference. Interference may negatively influence rather than improve outcomes when multiple techniques are applied (e.g., one may speculate that the freezing behavior in HRT may impede the accelerated behavior in DC, thus potentially confusing patients with different rationales).

## Methods

### Sample

The study was set up as a randomized controlled trial (no stratification). All assessments were carried out online using Questback/Unipark®. In keeping with the guidelines of the European general data protection regulation (GDPR), the online survey did not store IP addresses. There was no direct therapeutic contact/exchange between participants and researchers. Participation was anonymous and participants were instructed on how to create anonymous email addresses.

The study was posted on Facebook forums and topic-related German websites as an unguided treatment study for individuals with BFRBs. Individuals were randomly allocated to one of three treatment conditions or to a waitlist control group (allocation: 1:1:1:1). The three treatment conditions only differed in the order that manuals were received. Inclusion criteria were to be between 18 and 75 years old with at least one current self-reported BFRB. All genders were included. As the study was conducted in German, sufficient command of the German language was required. Concurrent treatments (e.g., medication, other self-help interventions, and psychotherapy) were tolerated. The trial was registered with the German Clinical Trials Register (DRKS00024526). Ethical approval was obtained from the local ethics committee for psychologists at the University Hospital of Hamburg-Eppendorf (Germany, LPEK-0249). The study was not externally funded. Sample size calculation relied on g*power; to detect a medium effect size at an alpha-level of 0.05 and a beta of 0.8 a sample size of 80 individuals per condition was required.

The sample of the present study did not overlap with other treatment studies on decoupling published by the authors. As can be seen in Table 1, participants were mainly female in their early 30s with few being prescribed psychotropic medication. More than half had suffered from skin picking and nail biting in their lifetime, while approximately 2/5 reported trichotillomania or LCB. Approximately half of the sample reported another psychiatric diagnosis. On average, participants had previously engaged in one psychotherapeutic treatment. While the study included participants with subclinical forms of BFRBs, few reported no acute somatic impairment (4.8%) or no impairment in social and occupational functioning (19.2%) according to the GBS-9.

**Table 1:** Demographic and psychopathological characteristics at baseline

	WLC (*n* = 85)	HRT (*n* = 81)			DC (*n* = 84)		DC-is (*n* = 84)	Statistics
*Demographic and background variables *								
Age in years	34.38 (10.20)	32.93 (9.80)			33.02 (11.27)		32.48 (10.86)	*F*(3, 330) = 0.51, *p* = 0.676, η_p_2 = 0.005
Gender (female/male/diverse)	70/15/0	65/15/1			71/13/0		67/17/0	χ^2^(6) = 3.82, *p* = 0.701
Prior psychotherapeutic treatments	1.27 (1.71)	0.80 (1.42)			1.37 (4.63)		0.84 (1.19)	*F*(3, 320) = 0.99, *p* = 0.298, η_p_2 = 0.009
*Medication*								
Antidepressants	14.1%	4.9%			10.7%		9.5%	χ^2^(3) = 4.01, *p* = 0.260
Antipsychotics	2.4%	2.5%			0%		0%	χ^2^(3) = 4.10, *p* = 0.251
Antiepileptics	0%	0%			0%		2.4%	χ^2^(3) = 5.99, *p* = 0.112
Other psychotropic substances	3.5%	1.2%			2.4%		1.2%	χ^2^(3) = 1.51, *p* = 0.679
No psychotropic substances	82.4%	93.8%			86.9%		89.3%	χ^2^(3) = 5.41, *p* = 0.144
*Generic BFRB Scale-9 (GBS-9) *								
Total score	16.01 (5.25)	15.70 (5.91)			16.06 (4.98)		14.70 (5.16)	*F*(3, 330) = 1.19, *p* = 0.313, η_p_2 = 0.011
Severity	9.64 (2.61)	9.59 (3.14)			9.45 (2.65)		9.23 (2.72)	*F*(3, 330) = 0.36, *p* = 0.782, η_p_2 = 0.003
Impairment	6.36 (3.21)	6.11 (3.46)			6.62 (3.17)		5.47 (3.06)	*F*(3, 330) = 1.98, *p* = 0.116, η_p_2 = 0.018
Depressive symptoms (PHQ-9)	8.16 (4.71)	8.11 (4.60)			9.11 (4.88)		8.02 (5.25)	*F*(3, 330) = 0.91, *p* = 0.437, η_p_2 = 0.008
Quality of life global item (WHOQOL-BREF)	3.52 (0.87)	3.78 (0.84)			3.37 (0.76)		3.52 (0.92)	*F*(3, 330) = 3.28, *p* = 0.021, η_p_2 = 0.029 [HRT > WLC: *p* = 0.049, *d* = 0.30; HRT > DC: *p* = 0.002, *d* = 0.51; HRT > DC-is: *p* = 0.056, *d* = 0.29]
*Lifetime prevalence BFRBs *								
Skin picking	54.1%	56.8%			59.5%		66.7%	χ^2^(3) = 3.05, *p* = 0.384
Nail biting	58.8%	58%			52.4%		67.9%	χ^2^(3) = 4.28, *p* = 0.233
Trichotillomania	42.4%	37%			42.9%		38.1%	χ^2^(3) = 0.90, *p* = 0.825
Lip-cheek biting	37.6%	39.5%			40.5%		36.9%	χ^2^(3) = 0.29, *p* = 0.962
Other	11.8%	13.6%			8.3%		7.1%	χ^2^(3) = 2.42, *p* = 0.490
*Current presence of BFRBs*								
Skin picking	47.1%	53.1%			52.4%		58.3%	χ^2^(3) = 2.16, *p* = 0.539
Nail biting	41.2%	35.8%			35.7%		44%	χ^2^(3) = 1.79, *p* = 0.618
Trichotillomania	37.6%	35.8%			35.7%		33.3%	χ^2^(3) = 0.34, *p* = 0.951
Lip-cheek biting	23.5%	25.9%			28.6%		31%	χ^2^(3) = 1.32, *p* = 0.724
Other	9.4%	11.1%			6%		6%	χ^2^(3) = 2.23, *p* = 0.526
*Lifetime prevalence of psychiatric disorders *								
No psychiatric disorder	45.9%	53.1%			48.8%		58.3%	χ^2^(3) = 2.97, *p* = 0.396
Depression	34.1%	23.5%			33.3%		25%	χ^2^(3) = 3.70, *p* = 0.295
Obsessive-compulsive disorder	18.8%	8.6%			14.3%		8.3%	χ^2^(3) = 5.76, *p* = 0.124
Anxiety disorder	14.1%	12.3%			14.3%		10.7%	χ^2^(3) = 0.64, *p* = 0.888
Posttraumatic stress disorder	4.7%	6.2%			4.8%		2.4%	χ^2^(3) = 1.43, *p* = 0.699
Substance or alcohol abuse	2.4%	0%			3.6%		2.4%	χ^2^(3) = 2.69, *p* = 0.443
Other	20%	13.6%			10.7%		16.7%	χ^2^(3) = 3.12, *p* = 0.373

*DC* decoupling, *DC-is* decoupling in sensu, *HRT* habit reversal training, *PHQ-9* patient health questionnaire-9 (depression), *WHOQOL-BREF* WHO quality of life – BREF, *WLC* waitlist control group

### Invitation, Baseline and Post-Assessment (Self-Report)

All participants provided electronic informed consent following an explanation of the study rationale. As an incentive for participation, all participants received another technique upon completion of the post-assessment (COGITO, a free app developed by our group to decrease psychological problems, especially low self-esteem and mood: www.uke.de/cogito_app). The waitlist control group also received the three manuals upon completion of the post-assessment.

Participants then answered questions on age, gender, psychopathology and treatment. Individuals were asked to endorse whether they had ever (i.e., lifetime prevalence) suffered from skin-picking, nail-biting, tearing out the hair (i.e., trichotillomania), biting the skin (or mouth) or other BFRBs resulting in either injuries, visible consequences (e.g., gnawed fingernails) or disabilities in social, occupational or other important areas of life (participants could endorse multiple options). These questions were repeated but with the time frame restricted to the last two weeks. BFRBs had to be named/described and data were only included if a known BFRB was disclosed. Most participant responses referred to joint cracking, dermatophagia and trichophagia. The next items related to the medical history (e.g., number of psychotherapeutic treatments, see Table 1). The generic body-focused repetitive behavior scale-9 (GBS-9), the WHO Quality of Life-BREF (WHOQOL-BREF; global item) and the patient health questionnaire (PHQ-9) were then administered (see below). Finally, we asked participants whether all questions had been answered truthfully and requested an anonymous email address.

**Fig. 1:**
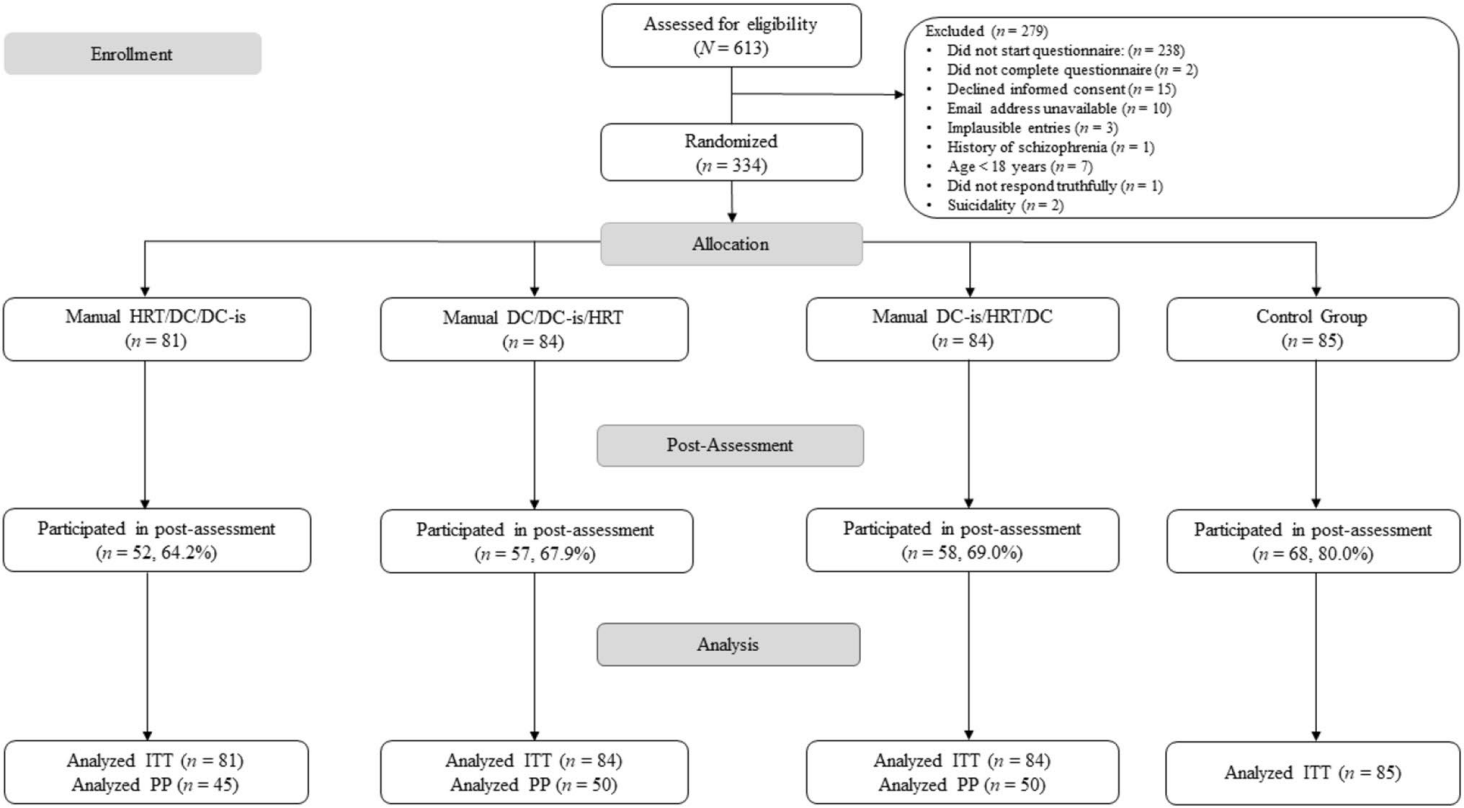
CONSORT flow chart

Participants were then automatically randomized, based on the date, to one of the four conditions (Fig. 1); participants in the experimental conditions were directed to a website to download the manuals as one zip-file. Thus, treatment allocation was concealed. Six weeks following the baseline assessment, all participants received automated emails inviting them to complete the post-assessment (up to three reminders were sent). For the post assessment, participants were first asked to enter the email address they had initially provided, and then completed the GBS-9, WHOQOL-BREF, and the PHQ-9 (reference time-frame: last week). We inquired about changes in therapeutic status, or about whether other self-help interventions/treatments had been applied over the course of study participation.

Individuals then had to report whether they were allocated to one of the treatment conditions or to the waitlist control group. Note that allocation was objective; this question verified whether participants were aware of allocation in the event that emails containing allocation information failed to be received, potentially leading participants to think they were in the control group. If allocated to a treatment condition, participants were asked to what degree they had used the manual, which was measured on a 7-point scale (not read at all; partially read one or more manuals but never used; used technique once in the last six weeks; used technique once weekly; used technique multiple times weekly; used technique on a daily basis; used technique several times daily). For those who indicated that they had at least started to read one of the manuals, we posed further questions related to comprehensibility, satisfaction and subjective efficacy of the techniques as well as the frequency of administration (see below). We also asked participants what they had liked or disliked about the study/the intervention techniques (optional). We again asked whether prior responses were made truthfully (yes, no). Before terminating, control participants had the option to download all manuals as well as the COGITO app.

### Primary Outcome

The Generic BFRB Scale-9 (GBS-9; Moritz et al., 2021) represented the primary outcome. The scale builds upon the revised Skin Picking Scale (SPS-R; Gallinat et al., 2001; Snorrason et al., 2012). The SPS-R has acceptable reliability as well as high convergent validity with the skin picking impact scale (SIPS; Keuthen et al., 2001; Mehrmann et al., 2017). The GBS-9 slightly reformulates the SPS-R to capture different forms of BFRBs. Participants had to provide a joint rating in the event that they suffered from different BFRBs (for further details see Moritz et al., 2021). Every item had to be rated on a 5-point Likert scale (range: 0–4). Similar to the German adaptation of the SPS-R and in keeping with the Massachusetts general hospital (MGH) Hairpulling Scale (Keuthen et al., 1995), control over the behavior was differentiated into control over the urge, and control over the behavior (the mean of the two scores was used as an estimate for control). For the baseline assessment, the time frame was set to the last two weeks; for the post assessment it was set to the last week. The items were as follows: (1) Frequency of the urge to perform BFRB (*from* 0 = no urge *to* 4 = constant urge (> 8 h per day)), (2) Intensity of the urge to perform BFRB (*from* 0 = no urge *to* 4 = extremely strong urge), (3) Control over the urge (*from* 0 = no urge or could always control urge *to* 4 = urge could not or only hardly be resisted), (4) Time spent performing BFRB (*from* 0 = no behavior *to* 4 = behavior performed almost constantly (> 8 h per day)), (5) Control over the BFRB (*from* 0 = full control: could always resist or terminate *to* 4 = no control, can never stop); (6) Emotional distress/suffering because of BFRB (*from* 0 = not at all *to* 4 = very high emotional distress, self-injurious behavior stressed me a lot), (7) Impairment in social and occupational functioning (*from* 0 = no impairment *to* 4 = extreme impairment), (8) Avoidance (*from* 0 = no avoidance *to* 4 = almost constant avoidance), (9) Acute somatic consequences (0 = no injuries *to* 4 = very severe injuries). Following recommendations for the SPS-R (Snorrason et al., 2012), we calculated two subscales as well as a total score. The symptom severity subscale was composed of the sum of items 1, 2, 4 and the mean rating of items 3 and 5. The impairment subscale was composed using the sum of items 6–9. In line with the pilot study (Moritz et al., 2021), a positive treatment response was defined as a decline of 35% on the primary outcome, in accordance with other studies on BFRBs (e.g., Grant et al., 2017).

The test-retest reliability of the GBS is satisfactory (*r* = 0.76, *p* < 0.001) (Moritz et al., 2021). For the present study, the test-retest reliability of the GBS-9 (control group) was good for the total score (*r* = 0.844, *p* < 0.001), severity subscale (*r* = 0.802, *p* < 0.001) and the impairment subscale (*r* = 0.812, *p* < 0.001). Cronbach’s alpha was good for all scales: total score (*α* = 0.857), severity (*α* = 0.812) and impairment (*α* = 0.826). In an independent study (Moritz et al., 2022), we validated an eight item version of the GBS (one control item instead of two as in the GBS-9) against the repetitive body focused behavior scale (RBFBS, self-report) (Selles et al., 2018), adapted for adults, in 279 individuals with BFRBs showing satisfactory convergent validity (*r* = 0.74, *p* < 0.001).

### Secondary Outcomes

We used the global item (“How would you assess your quality of life?“; response options: *very poor* (= 1) to *very good* (= 5)) on the WHOQOL-BREF (Skevington et al., 2004) as an index for quality of life (reference: last 2 weeks). The PHQ-9 (Gilbody et al., 2007; Kroenke et al., 2001) assessed depressive symptoms and matches the nine diagnostic criteria for depression according to DSM-IV (scoring: not at all (= 0), several days (= 1), more than half the days (= 2), nearly every day (= 3). The scale is derived from the primary care evaluation of mental disorders (PRIMEMD); its psychometric properties are good (Gilbody et al., 2007).

### Subjective Appraisal and Benefit

Participants who had at least begun to read the manual were asked to rate (each of the three techniques separately) to which degree it had helped them (not at all, somewhat, noticeable, very much, *not performed*). We then asked whether participants had employed the manuals in parallel or in succession. We also asked participants how many days they had practiced each technique during the last 6 weeks. Further, items from the Patient Satisfaction Questionnaire (CSQ-8; German version by Schmidt et al., 1989) were adapted for online interventions and assessed the subjective appraisal of each of the techniques separately (e.g., quality, satisfaction, subjective efficacy, intention to use the application in the future). Table 2 shows results of the CSQ-8 items as well as additional questions from the Subjective Efficacy Scale for each intervention (e.g., Moritz et al., 2019). Questions were posed only if a technique was applied.

**Table 2:** Assessment of the per protocol sample on the individual techniques

Item	HRT first (HRT)	DC first (DC)	DC-is first (DC-is)	Statistics [Group differences in square brackets]
1. I think the manual is good for self-help and self-guidance	3.08 (0.85) [95.7%]	2.87 (0.98) [93.4%]	2.76 (1.03) [87.3%]	*F*(1.72, 104.74) = 3.80, *p* = 0.032, η_p_^2^ = 0.059, [HRT > DC-is: *p* = 0.008, *d* = 0.36]
2. My symptoms decreased because of the application of the program	1.98 (0.93) [68.5%]	1.87 (0.88) [67%]	1.65 (0.83) [50.7%]	*F*(2, 122) = 3.86, *p* = 0.024, η_p_^2^ = 0.059, [DC > DC-is: *p* = 0.090, *d* = 0.25; HRT > DC-is: *p* = 0.016, *d* = 0.36]
3. I think the content of the manual was comprehensible	3.56 (0.67) [98.9%]	3.40 (0.76) [96.7%]	3.34 (0.74) [100%]	*F*(1.75, 106.67) = 3.99, *p* = .026, η_p_^2^ = 0.061, [HRT > DC: *p* = .007, *d* = 0.24; HRT > DC-is: *p* = .018, *d* = 0.31]
4. I think the manual was helpful	3.06 (0.94) [95.7%]	2.89 (0.99) [92.3%]	2.60 (0.98) [90.1%]	*F*(2, 122) = 6.55, *p* = 0.002, η_p_^2^ = 0.097, [DC > DC-is: *p* = 0.033, *d* = 0.27; HRT > DC-is: *p* = 0.001, *d* = 0.48]
5. I was able to use the manual on a regular basis during the past 6 weeks	1.98 (0.88) [60.9%]	2.02 (0.84) [70.3%]	1.84 (0.87) [59.2%]	*F*(2, 122) = 0.96, *p* = 0.385, η_p_^2^ = 0.016
6. I had to force myself to use the manual	2.65 (1.07) [84.8%]	2.50 (1.07) [81.3%]	2.65 (1.10) [83.1%]	*F*(2, 122) = 0.87, *p* = 0.422, η_p_^2^ = 0.014
7. The manual is not applicable to my behavior	1.52 (0.86) [31.5%]	1.55 (0.93) [27.5%]	1.97 (1.13) [46.5%]	*F*(1.58, 96.39) = 6.78, *p* = 0.004, η_p_^2^ = 0.100, [DC-is > DC: *p* = 0.014, *d* = 0.39; DC-is > HRT: *p* = 0.001, *d* = 0.46]
8. I have performed fewer behaviors (e.g., nail biting, hair pulling, etc.) because of the manual	2.03 (0.89) [69.6%]	1.79 (0.77) [67%]	1.71 (0.84) [56.3%]	*F*(2, 122) = 3.69, *p* = 0.028, η_p_^2^ = 0.057, [HRT > DC-is: *p* = 0.009, *d* = 0.36]
9. Would you recommend the manual to a friend with similar symptoms?	2.92 (0.91) [93.5%]	2.95 (0.91) [93.4%]	2.73 (1.09) [84.5%]	*F*(1.81, 110.72) = 2.28, *p* = 0.112, η_p_^2^ = 0.036
10. Would you use the manual again?	2.84 (1.01) [90.2%]	2.71 (1.11) [85.7%]	2.32 (1.21) [67.6%]	*F*(2, 122) = 5.10, *p* = 0.007, η_p_^2^ = 0.077, [DC > DC-is: *p* = 0.023, *d* = 0.32; HRT > DC-is: *p* = 0.003, *d* = 0.46]

Response options: 1 = does not apply at all, 2 = applies somewhat, 3 = rather applies, 4 = fully applies. In square brackets, the frequency of endorsement is displayed (options 2–4)

*DC* decoupling, *DC-is* decoupling in sensu, *HRT* habit reversal training

### Intervention

Three intervention conditions were set up, which only differed in the order manuals were dispatched as pdf-files (condition 1: HRT/DC/DC-is; condition 2: DC/DC-is/HRT; condition 3: DC-is/HRT/DC; aggregated as zip-files). Each manual was placed in either first, second or third order. All manuals are available at no cost via www.free-from-bfrb.org.

The HRT technique (8 pages) followed the classical description by Azrin & Nunn (1973). First, the phenomenology of different BFRBs was described as well as the possible somatic and psychological consequences of BFRBs. The protocol taught the two main components of HRT according to dismantling studies: (1) Awareness training: the participants had to identify and note situations that typically trigger BFRBs as well as times when BFRBs were most prevalent. (2) Competing response training: participants were instructed to perform antagonistic (static) behaviors for 1–3 min once an urge to engage in BFRBs was noticed, or as a means to stop an ongoing BFRB (e.g., clenching first, sitting on hands, folding hands). Several examples of how to conduct the technique were provided and illustrated with photos.

For the DC technique (12 pages), the introduction was virtually identical to the HRT manual. Participants were then instructed on how to shape/deviate their dysfunctional behavior into a similar but benign movement. Two steps were distinguished. In the initiating phase of DC, the movement had to be like the dysfunctional behavior. Shortly before reaching the prior behavioral target (e.g., nails), that is, in temporal and spatial proximity, the movement should be deviated and target either another location on the body (e.g., ear) or a certain point in the room with *an accelerated movement*. Instructions were illustrated by photos. Participants were instructed on how to use a smartphone timer to remind them to complete the exercises (a timer was encouraged as DC should be practiced in both symptomatic and symptom-free intervals, which is different to HRT where the new behavior is usually only executed in symptomatic periods that often do not follow exact time periods).

The Decoupling in sensu (DC-is) technique (8 pages) employed the same introduction. Unlike DC, which is executed at an entirely behavioral level, in DC-is the movement of the initiating phase should be *imagined*. Shortly before performing the imagined BFRB (e.g., biting nails), the imagined movement is interrupted by an actual movement. Here, the hand, in the case of nail biting for example, that was previously imagined, should in reality then be moved away from the body with the fingers spread wide (the hand was clenched into a fist during the first phase). In other words, the imagined sequence is terminated by a behavioral counter-response. The revised DC protocol is intended to allow for greater generalization than conventional DC (Moritz & Rufer et al., 2020). We also encouraged using a smartphone timer. The main difference in the competing response of HRT compared to the DC techniques is that in the former method a static (“freezing”) behavior is performed when the urge is felt/BFRBs are executed. DC and DC-is are dynamic responses that are practiced in symptom-free intervals and are targeted at the urge and aimed to prevent the initiation of the movement itself.

### Data Analysis Strategy

Main results were computed with 4 × 2 two-way ANOVAs with condition as the between-group factor and time (baseline, post) as the within-subject factor. Data from all participants were considered in intention-to-treat (ITT) analyses. Missing data were imputed using expectation-maximization. Per protocol analyses considered participants with complete data, under the additional condition that participants in the experimental groups must have read (PP read) or performed (PP performed) the exercises. We also looked at a subgroup who had performed the exercises regularly according to self-report (PP frequent). The level of statistical significance was set at *p* < 0.05 for all analyses.

## Results

The four groups (*N* = 334) did not differ on any major sociodemographic or psychometric baseline variable except for slightly higher quality of life in the HRT group (see Table 1). Almost half the sample endorsed more than one BFRB (1 current BFRB: 53.6%, 2 BFRBs: 30.5%, 3 BFRBs: 13.5%, 4 BFRBs in 2.4%).The mean number of BFRBs was 1.61 (SD = 0.85).

For the main analyses mixed ANOVAs with group (conditions) as the between-subject factor and time (pre, post) as the within-subject factor were conducted.

Table 3 shows that the GBS-9 total score significantly declined across all conditions, including the waitlist control (WLC). Yet, decline was significantly steeper for all treatment conditions compared to the WLC (ITT, PP read, PP performed). For most analyses, medium or medium-to-large effect sizes were observed for all treatment conditions relative to the WLC. For PP frequent, the difference between HRT-first and WLC failed to reach significance. Similar results emerged for the GBS-9 impairment and severity scales, with a clear advantage for DC and DC-is-first over the WLC. For PP frequent, again there was no difference between HRT-first and the WLC. Outcome on the impairment subscale was also significantly poorer in HRT-first compared to DC-first. For quality of life, paired t-tests showed significant improvement only for DC-first, and group differences were significant compared to the WLC and HRT-first conditions. Regarding depressive symptoms, paired t-tests showed improvement for HRT and DC-first, whereas a trend was found for DC-is-first. Only DC-first showed a significantly greater decline in comparison to the WLC group. For some analyses, DC-first was superior to HRT-first (quality of life: all analyses, depression: PP read, PP performed, PP frequent) and DC-is-first (depressive symptoms: PP read). In subsidiary analyses, we compared the three conditions against each other for each BFRB subtype separately on the primary outcome. No significant differences emerged (*p* > 0.1).

**Table 3:** Complete cases, per protocol and intention-to-treat analyses across time for primary and secondary outcomes

	WLC (*n* = 68)		HRT first (HRT; *n* = 52)		DC first (DC; *n* = 57)		DC-is first (DC-is; *n* = 58)		Intention to treat (ITT) (WLC: *n* = 85; HRT first: *n* = 81; DC first: *n* = 84; DC-is first: *n* = 84)	PP read: at least read manual (WLC: *n* = 68, HRT: *n* = 45, DC: *n* = 50; DC-is: *n* = 50)		PP performed: at least once performed exercises (WLC: *n* = 68, HRT: *n* = 33, DC: *n* = 40; DC-is: *n* = 35)		PP frequent: technique(s) performed at least once weekly (WLC: *n* = 68, HRT: *n* = 22, DC: *n* = 24; DC-is: *n* = 18)	
	Pre	Post	Pre	Post	Pre	Post	Pre	Post		T = Time; I = Interaction; differences across conditions in square brackets					
GBS-9 total	15.62 (5.44)	14.18 (5.13) [*p* < 0.001]	15.06 (5.55)	11.57 (6.17) [*p* < 0.001]	15.48 (4.59)	11.92 (5.42) [*p* < 0.001]	15.40 (5.17)	11.76 (5.39) [*p* < 0.001]	T: *F*(1, 330) = 225.91, *p* < 0.001, η_p_^2^ = 0.406 I: *F*(3, 330) = 4.87, *p* = 0.002, η_p_^2^ = 0.042 [HRT > WLC: *p* = 0.003, *d* = 0.54; DC > WLC: *p* = 0.001, *d* = 0.52; DC-is > WLC: *p* = 0.002, *d* = 0.52]	T: *F*(1, 209) = 155.03, *p* < 0.001, η_p_^2^ = 0.426 I: *F*(3, 209) = 6.57, *p* < 0.001, η_p_^2^ = 0.086 [HRT > WLC: *p* = 0.002, *d* = 0.68; DC > WLC: *p* = 0.001, *d* = 0.56; DC-is > WLC: *p* < 0.001, *d* = 0.83]		T: *F*(1, 172) = 144.81, *p* < 0.001, η_p_^2^ = 0.457 I: *F*(3, 172) = 8.46, *p* < 0.001, η_p_^2^ = 0.129 [HRT > WLC: *p* = 0.009, *d* = 0.63; DC > WLC: *p* < 0.001, *d* = 0.77; DC-is > WLC: *p* < 0.001, *d* = 0.93]		T: *F*(1, 128) = 101.15, *p* < 0.001, η_p_^2^ = 0.441 I: *F*(3, 128) = 8.19, *p* < 0.001, η_p_^2^ = 0.161 [HRT > WLC: *p* = 0.079, *d* = 0.45; DC > WLC: *p* < 0.001, *d* = 0.94; DC-is > WLC: *p* = 0.001, *d* = 0.96; DC > HRT: *p* = 0.060, *d* = 0.49]	
GBS-9 severity	9.52 (2.78)	8.40 (2.85) [*p* < 0.001]	9.25 (3.05)	7.16 (3.38) [*p* < 0.001]	9.24 (2.34)	7.25 (3.00) [*p* < 0.001]	9.61 (2.60)	7.46 (2.76) [*p* < 0.001]	T: *F*(1, 330) = 235.15, *p* < 0.001, η_p_^2^ = 0.416 I: *F*(3, 330) = 2.48, *p* = 0.061, η_p_^2^ = 0.022 [HRT > WLC: *p* = 0.016, *d* = 0.41; DC > WLC: *p* = 0.029, *d* = 0.34; DC-is > WLC: *p* = 0.015, *d* = 0.41]	T: *F*(1, 209) = 152.88, *p* < 0.001, η_p_^2^ = 0.422 I: *F*(3, 209) = 3.82, *p* = 0.011, η_p_^2^ = 0.052 [HRT > WLC: *p* = 0.014, *d* = 0.53; DC > WLC: *p* = 0.026, *d* = 0.41; DC-is > WLC: *p* = 0.003, *d* = 0.63]		T: *F*(1, 172) = 143.06, *p* < 0.001, η_p_^2^ = 0.454 I: *F*(3, 172) = 4.89, *p* = 0.003, η_p_^2^ = 0.079 [HRT > WLC: *p* = 0.021, *d* = 0.55; DC > WLC: *p* = 0.003, *d* = 0.56; DC-is > WLC: *p* = 0.002, *d* = 0.72]		T: *F*(1,128) = 91.46, *p* < .001, η_p_^2^ = 0.417 I: *F*(3, 128) = 3.76, *p* = 0.012, η_p_^2^ = 0.081 [HRT > WLC: *p* = 0.085, *d* = 0.42; DC > WLC: *p* = 0.011, *d* = 0.59; DC-is > WLC: *p* = 0.012, *d* = 0.72]	
GBS-9 impairment	6.10 (3.19)	5.78 (3.06) [*p* = 0.200]	5.81 (3.18)	4.40 (3.27) [*p* < 0.001]	6.25 (3.08)	4.67 (2.96) [*p* < 0.001]	5.79 (3.23)	4.14 (2.87) [*p* < 0.001]	T: *F*(1, 330) = 99.01, *p* < 0.001, η_p_^2^ = 0.231 I: *F*(3, 330) = 4.71, *p* = 0.003, η_p_^2^ = 0.041 [HRT > WLC: *p* = 0.010, *d* = 0.44; DC > WLC: *p* < 0.001, *d* = 0.53; DC-is > WLC: *p* = 0.005, *d* = 0.46]	T: *F*(1, 209) = 71.70, *p* < 0.001, η_p_^2^ = 0.255 I: *F*(3, 209) = 5.56, *p* = 0.001, η_p_^2^ = 0.074 [HRT > WLC: *p* = 0.006, *d* = 0.57; DC > WLC: *p* = 0.001, *d* = 0.55; DC-is > WLC: *p* = 0.001, *d* = 0.73]	T: *F*(1, 172) = 64.42, *p* < 0.001, η_p_^2^ = 0.272 I: *F*(3, 172) = 7.05, *p* < 0.001, η_p_^2^ = 0.110 [HRT > WLC: *p* = 0.043, *d* = 0.46; DC > WLC: *p* < 0.001, *d* = 0.75; DC > HRT: *p* = 0.094, *d* = 0.35; DC-is > WLC: *p* = 0.001, *d* = 0.79]		T: *F*(1, 128) = 47.06, *p* < 0.001, η_p_^2^ = 0.269 I: *F*(3, 128) = 7.71, *p* < 0.001, η_p_^2^ = 0.153 [DC > WLC: *p* < 0.001, *d* = 0.92; DC-is > WLC: *p* = 0.002, *d* = 0.89; DC > HRT: *p* = 0.017, *d* = 0.65; DC-is > HRT: *p* = 0.075, *d* = 0.59]		
Quality of life global item (WHOQOL-BREF)	3.54 (0.78)	3.59 (0.70) [*p* = 0.634]	3.77 (0.83)	3.67 (0.90) [*p* = 0.374]	3.37 (0.79)	3.77 (0.82) [*p* < 0.001]	3.54 (0.93)	3.70 (0.94) [*p* = 0.162]	T: *F*(1, 330) = 9.22, *p* = 0.003, η_p_^2^ = 0.027 I: *F*(3, 330) = 5.67, *p* = 0.001, η_p_^2^ = 0.049 [DC > WLC: *p* = 0.011, *d* = 0.39; DC > HRT: *p* < 0.001, *d* = 0.64; DC-is > HRT: *p* = 0.029, *d* = 0.33; DC > DC-is: *p* = 0.089, *d* = 0.28]	T: *F*(1, 209) = 5.68, *p* = 0.018, η_p_^2^ = 0.026 I: *F*(3, 209) = 2.94, *p* = 0.034, η_p_^2^ = 0.040 [DC > WLC: *p* = 0.022, *d* = 0.45; DC > HRT: *p* = 0.006, *d* = 0.60]	T: *F*(1, 172) = 8.22, *p* = 0.005, η_p_^2^ = 0.046 I: *F*(3, 172) = 2.26, *p* = 0.083, η_p_^2^ = 0.038 [DC > WLC: *p* = 0.035, *d* = 0.43; DC > HRT: *p* = 0.043, *d* = 0.47]		T: *F*(1, 128) = 6.70, *p* = 0.011, η_p_^2^ = 0.050 I: *F*(3, 128) = 2.52, *p* = 0.061, η_p_^2^ = 0.056 [DC > WLC: *p* = 0.025, *d* = 0.53; DC > HRT: *p* = 0.028, *d* = 0.63]		
Depressive symptoms, (PHQ-9)	8.13 (4.85)	7.88 (4.44) [*p* = 0.546]	8.06 (4.55)	6.90 (4.90) [*p* = 0.013]	9.30 (4.67)	6.88 (5.14) [*p* < 0.001]	7.90 (4.86)	6.74 (5.36) [*p* = 0.087]	T: *F*(1, 330) = 32.75, *p* < 0.001, η_p_^2^ = 0.089 I: *F*(3, 330) = 3.00, *p* = 0.031, η_p_^2^ = 0.027 [DC > WLC: *p* = 0.003, *d* = 0.47]	T: *F*(1, 209) = 22.17, *p* < 0.001, η_p_^2^ = 0.096 I: *F*(3, 209) = 3.87, *p* = 0.010, η_p_^2^ = 0.053 [DC > WLC: *p* = 0.001, *d* = 0.63; DC > HRT: *p* = 0.041, *d* = 0.44; DC > DC-is: *p* = 0.044, *d* = 0.34]	T: *F*(1, 172) = 18.11, *p* < 0.001, η_p_^2^ = 0.095 I: *F*(3,172) = 3.92, *p* = 0.010, η_p_^2^ = 0.064 [DC > WLC: *p* = 0.001, *d* = 0.64; DC > HRT: *p* = 0.025, *d* = 0.52; DC > DC-is: *p* = 0.085, *d* = 0.34]		T: *F*(1, 128) = 21.59, *p* < 0.001, η_p_^2^ = 0.144 I: *F*(3, 128) = 5.42, *p* = 0.002, η_p_^2^ = 0.113 [DC > WLC: *p* < 0.001, *d* = 0.80; DC > HRT: *p* = 0.010, *d* = 0.71; DC-is > WLC: *p* = 0.064, *d* = 0.53]		

*DC* decoupling, *DC-is* decoupling in sensu, *GBS-9* generic body-focused repetitive behavior scale-9, *HRT* habit reversal training, *PHQ-9* patient health questionnaire 9 (depression), *WHOQOL-BREF* WHO quality of life – BREF, *WLC* waitlist control group

The median improvement on the GBS-9 was 9.5% in the WLC condition compared to 23.9% in the HRT-first condition (PP read: 27.8%), 20% in the DC-first condition (PP read: 23.4%) and 23.9% in the DC-is-first condition (PP read: 25.7%). Improvement by at least 35% on the GBS-9 total score was displayed by 7.4% of the WLC condition, 30.8% of the HRT-first condition, 33.3% of the DC-first condition and 27.6% of the DC-is-first condition (complete cases sample).

We explored whether differences were reflected by greater usage of the different techniques, for (1) days using the technique(s), and (2) utilization behavior (parallel or sequentially) in the DC-first condition. No significant differences emerged (*F* < 1.3, *p* > 0.2 for all comparisons, before correcting for multiple comparisons).

Regarding assessment of individual techniques, all interventions received very favorable ratings (> 80%) for items 1 (good for self-help), 3 (comprehensible), 4 (helpful) and 9 (recommendation). DC and/or HRT received more favorable results than DC-is on items 1, 2 (symptoms decreased), 3, 4, 7 (manual not applicable), 8 (symptoms decreased) and 10 (would use manual again). Approximately 2/3 of individuals reported their symptoms had decreased due to HRT and DC, while only half endorsed this for DC-is (item 2). We also assessed whether DC was rated inferior relative to HRT in those with skin picking, as shown in a prior study (Moritz et al., 2012), which was not the case. On item 2, DC (*M* = 2.11, *SD* = 0.83) was rated numerically even better than HRT by this subtype (*M* = 1.93, *SD* = 0.90) but failed to reach the conventional threshold of significance (*p* = 0.105).

### Additive Effects

We correlated the number of techniques applied (0–3) in the PP read sample with symptom improvement on the GBS-9. A small but significant correlation emerged (*r* = 0.280, *p* = 0.001). As one may argue that effects may reflect greater adherence and perhaps more practice with more techniques applied, we corrected for daily use. Results were corroborated (*r* = 0.245, *p* = 0.005). Symptom decline on the GBS-9 total score was greater for those in the experimental groups using all three techniques (*n* = 63; *M* = 5.07, *SD* = 0.4.37), followed by two techniques (*n* = 20; *M* = 4.00, *SD* = 3.16), one technique (*n* = 26; *M* = 2.81, *SD* = 3.52) and no technique (*n* = 20; *M* = 2.37, *SD* = 2.95; significant post-hoc differences between those applying 3 techniques versus 1 (*p* = 0.013) or 0 techniques (*p* = 0.007) used).

## Discussion

This study set out to compare the efficacy of three different techniques for BFRBs, conveyed to participants in a different order, against a waitlist control group. We were also interested in subjective feasibility and efficacy and whether results extend beyond the primary symptoms of BFRBs and reach depressive symptoms and quality of life.

Results show that the application of different behavioral techniques led to substantial improvement of BFRBs. Interestingly, the more participants applied the techniques, the greater the improvement on the GBS-9. This finding speaks to an add-on effect. The differential improvement showed a stepwise function with those using all three manuals showing significantly greater improvement than those using only one manual. No differences were observed for those using the technique in parallel or sequentially. All three treatment groups, which only differed in the order the manuals were delivered, were superior to the waitlist control group on the primary outcome (GBS-9, except for HRT vs. WLC for the PP frequent condition). Interestingly, the DC-first condition (i.e., DC/DC-is/HRT) was superior relative to the waitlist control group for all ITT and PP analyses for depressive symptoms and quality of life. In contrast, the other treatment conditions did not show a clear advantage relative to the waitlist control group on secondary outcomes. For some analyses, HRT-first performed significantly worse than DC-first (e.g., quality of life). This may be due to a regression to the mean in the HRT-first condition, as quality of life was highest in this group at baseline. Future studies should examine whether the superiority of DC-first on the secondary (affective) outcomes reflects a primary effect on well-being or is owing to an indirect effect of improved impairments under DC on depression/quality of life (a post-hoc analysis showed that the GBS-9 impairment subscale was more strongly related to quality of life (*r* = 0.46) and depression (*r* = − 0.48) than the severity subscale (depression, *r* = 0.28; quality of life, *r* = − 0.29).

Importantly, the superiority of the DC-first effect was not due to greater usage of manuals in this condition: individuals neither spent more days using the techniques nor used more techniques. Subjective appraisal was most favorable for the DC and HRT techniques. Findings thus corroborate prior results indicating the efficacy of the three techniques when used as a self-help treatment (Moritz & Rufer 2011, 2020;  Moritz et al., 2011, 2021; Weidt et al., 2015). Unlike a prior study (Moritz et al., 2012), no evidence was found for lower efficacy of DC relative to HRT according to self-report for skin picking.

Strengths of the study include a large population sample and application of a new transdiagnostic scale. The low-threshold and self-help nature of the program is another important strength; people with BFRBs typically do not seek appropriate treatments due to shame and lack of availability of therapists, the latter being especially pertinent during the COVID-19 pandemic.

Several limitations warrant discussion. First, an active control group was not implemented. Future studies may choose psychoeducation or a technique with no known influence on BFRBs to account for placebo effects. We would also like to acknowledge that there are other competing response training protocols than the one used in the HRT condition; for example, competing response training can be conveyed with non-static methods (Franklin & Tolin, 2007; Sharenow et al., 1989; Woods et al., 1999) which, however, differ from decoupling. Of note, good effects can be achieved with HRT if tension is maintained for a shorter duration (Twohig & Woods, 2001). Additionally, awareness training usually involves social feedback, which due to the self-help nature of the trial could not be realized. Moreover, HRT is typically conveyed by a therapist, and thus the effect of HRT may be under-estimated by the use of a self-help technique. Further, the potential effects of DC and DC-is delivered by a therapist have not been evaluated. A head-to-head comparison of the three techniques guided by a therapist, and whether therapist-guided treatment results in greater improvement when compared to self-help, awaits examination. While the overall improvement rate was good (approximately 3/10 completers showed a 35% decline in BFRB symptoms) we do not know if these effects are sustained over time. Follow-up studies are needed to clarify this. Furthermore, studies may also investigate whether sequential usage (i.e., restricting access to manuals so that they are received and used one by one) shows different effects than parallel usage of the techniques. We would also like to acknowledge that our choice to include awake bruxism as a BFRB may be regarded controversial as the DSM-5 does not explicitly include it as a BFRB. Finally, unlike in the present study, we recommend using scales with the same retrospective time period for the baseline and subsequent assessments.

## Conclusion

This study has several important clinical implications in our opinion. Rather than providing participants with three different manuals with large overlap/redundancy on the introductory description of BFRBs and its consequences, we believe a synergistic approach that teaches the different techniques in a single manual is advantageous. Importantly, approximately 80% of participants reported that they had to force themselves to use the manuals, which also speaks to abbreviated treatments. This has already been done in the course of this study, and we now provide an integrated manual to participants at no cost (see www.uke.de/free-from-bfrb). The manual presents HRT first. In light of the present findings, we now encourage participants to start with DC and then try out other techniques. The recommendation to use techniques sequentially rather than in parallel will be removed.

Against the background of large heterogeneity pertaining to behavior (e.g., trichotillomania, skin picking), mode of action (focused or automatic), comorbid conditions (e.g., with and without depression), and individual preferences, treatments from different spheres (psychological, behavioral, sensory) may prove more effective by allowing individuals more flexibility than working solely on one aspect. Further, we recommend complementing behavioral approaches such as HRT, DC and DC-is with cognitive interventions aimed, for example, at acceptance, perfectionism, and self-esteem. To meet this aim we have recently developed a smartphone app (COGITO) that addresses emotional problems in a transdiagnostic fashion; two studies demonstrate the feasibility and efficacy of the approach for people with psychological problems (Bruhns et al., 2021; Lüdtke et al., 2018). Determining the most effective order of administration and whether these additions enhance outcomes awaits investigation.
